# Pirfenidone in Idiopathic Pulmonary Fibrosis: Real-World Observation on Efficacy and Safety, Focus on Patients Undergoing Antithrombotic and Anticoagulant

**DOI:** 10.3390/ph17070930

**Published:** 2024-07-11

**Authors:** Nicolò Reccardini, Maria Chernovsky, Francesco Salton, Paola Confalonieri, Lucrezia Mondini, Mariangela Barbieri, Antonio Romallo, Marta Maggisano, Chiara Torregiani, Pietro Geri, Michael Hughes, Corrado Campochiaro, Marco Confalonieri, Angelo Scarda, Umberto Zuccon, Barbara Ruaro

**Affiliations:** 1 Department of Pulmonology, University Hospital of Cattinara, 34149 Trieste, Italy; 2 Department of Medical, Surgical and Health Sciences, University of Trieste, 34127 Trieste, Italy; 3 Division of Musculoskeletal and Dermatological Sciences, Faculty of Biology, Medicine and Healt, The University of Manchester & Salford Royal NHS Foundation Trust, Manchester M6 8HD, UK; 4 Unit of Immunology, Rheumatology, Allergy and Rare Diseases (UnIRAR), IRCCS San Raffaele Hospital, 20132 Milan, Italy; 5 Pulmonology Unit, General Hospital “Santa Maria degli Angeli”, 33170 Pordenone, Italy

**Keywords:** idiopathic pulmonary fibrosis (IPF), pirfenidone, interstitial lung disease (ILD), antithrombotic treatment, anticoagulants, pulmonary function test (PFT)

## Abstract

Idiopathic pulmonary fibrosis (IPF) is a rare and progressive interstitial lung disease characterized by irreversible distortion of lung architecture and subsequent loss of pulmonary function. Pirfenidone is an antifibrotic agent associated with increased progression-free survival and overall survival rates, but it carries multiple side effects. The aim of the study was to examine the efficacy and safety profile of pirfenidone in a real-life context, with a focus on the concomitant use of antithrombotic and/or anticoagulant treatments. The clinical and functional data (forced vital capacity [FVC], forced expiratory volume in 1 s [FEV1], diffusing lung capacity for carbon monoxide [DLCO], and 6 min walking test distance [6MWD]) of all IPF patients treated with pirfenidone and referred to our two centers between 2019 and 2022 were retrospectively analyzed at baseline, 6 and 12 months after the start of treatment. A total of 55 IPF subjects undergoing pirfenidone treatment were included in the analysis (45.5% females, median [IQR] age at disease onset 68.0 [10.0] years, median [IQR] age at baseline 69.0 [10.8] years). Compared to baseline, at 12 months, FVC (86.0% vs. 80.0%; *p* = 0.023) and DLCO (44.0% vs. 40.0%; *p* = 0.002) were significantly reduced, while FEV1 (*p* = 0.304) and 6MWD (*p* = 0.276) remained stable; no significant change was recorded at 6 months. Most of the reported adverse events were mild or moderate. Gastrointestinal intolerance (9.1%) was the main cause of treatment discontinuation. A total of 5% of patients reported at least one minor bleeding event, although all episodes occurred in those receiving concomitant antithrombotic or anticoagulant. Overall, this real-life experience confirms the efficacy and safety profile of pirfenidone in the case of the concomitant use of antithrombotic and/or anticoagulant drugs.

## 1. Introduction

Idiopathic pulmonary fibrosis (IPF) is the most common type of interstitial lung disease (ILD), characterized by irreversible damage to the parenchyma, distortion of the lung architecture, significant reduction of the alveolar gas exchange capacity, and a median survival rate of 2–3 years from the moment of diagnosis. Although rare, with an annual incidence of 1.25–23.4 per 100,000 people in Europe, both its incidence and prevalence are increasing [1,2,3,4,5,6]. IPF onset is most commonly diagnosed in patients over 60 years of age and is predominantly affecting the male sex [7,8]. Symptoms of IPF are oftentimes nonspecific, ranging from exertion dyspnea to persistent dry coughing, which gradually develops over a period of several months or years, making initial diagnosis difficult. During physical examination, patients may present dry crackles, usually at the basilar segments of both lungs, indicative of parenchymal fibrosis in the affected areas. In later stages, respiratory failure, secondary pulmonary hypertension, and subsequent cor pulmonale may develop [9]. In September 2018, a joint statement led by the American Thoracic Society (ATS), redefined the diagnostic criteria for IPF, rendering the definite usual interstitial pneumonia (UIP) pattern presence in high-resolution computed tomography (HRCT) scans sufficient for diagnosis, with other patterns (such as probable UIP) requiring a compatible biopsy [10]. The UIP pattern must encompass the following criteria to be classified as definite: the presence of subpleural fibrosis predominantly in the basilar segments and often with heterogeneous distribution, honeycombing with or without traction bronchiectasis, and thickening of the interlobular septa which is often accompanied by the presence of a reticular pattern and limited areas of ground-glass type opacities. The probable UIP pattern follows the same characteristics but lacks the presence of honeycombing, which may appear at a later period in time and re-classify the probable UIP into a definite one. While the UIP pattern is typical of IPF, it is important to remember that other interstitial lung diseases (ILDs) may also present such a pattern, such as hypersensitivity pneumonia with fibrosis, stage IV sarcoidosis, or rheumatoid arthritis with pulmonary involvement [10,11,12,13]; in such cases, it is important to keep in mind the namesake of IPF, it is an idiopathic disease and as such all other potential diseases must be excluded prior to making a concrete diagnosis [14]. When discussing lung parenchyma damage in IPF, it is important to remember that it is not inflammation that is responsible for most of the damage, but rather the aberrant tissue repair caused by massive and dysregulated production of collagen by fibroblast foci, which create a three-dimensional net of fibrosis responsible for the UIP pattern typically observed in IPF. As such, therapeutic drugs aiming to inhibit inflammation have little success when treating IPF, rendering the disease incurable and difficult to control to this day [14,15,16]. Tracking the progression of IPF requires a combination of repeated pulmonary function tests, as well as clinical evaluation and HRTC scans every 6–12 months, depending on the severity of the disease. When referring to functional tests, forced vital capacity (FVC) and carbon monoxide diffusion capacity of the lung (DLCO) are the two main parameters upon which the progression of IPF is evaluated, as well as pulse oximetry both at rest and during exercise (such as the six-minute walking distance test) [16]. A cutoff value of >10% for FVC and of >15% of DLCO over a period of 12 months constitutes the value for disease progression [17,18,19]. Although IPF is unfortunately still considered an incurable disease, in the last two decades, novel therapies targeting the process of fibrosis itself have been approved for use in IPF: nintedanib and pirfenidone. Pirfenidone is an antifibrotic agent with a broad spectrum of action that is able to drastically decrease disease activity by targeting the mechanisms behind the formation of lung fibrosis itself [20]. The mechanism of action is not entirely understood as of yet but seems to target several pathways leading to fibroblast activation and the conversion of fibroblasts to myofibroblasts, such as α smooth muscle actin (α-SMA), collagen-I (Col-1), vimentin, hydroxyproline and interleukin 1β (IL-1β) and IL-18, as well as E-cadherin, transforming growth factor β (TGFβ) and tumor necrosis factor α (TNFα) in early intervention [20,21]. Perhaps the most interesting find, though, was its capability to inhibit the Janus Kinase 2/signal transducer and activator of transcription 3 (JAK2/STAT3) pathway, which leads to alternative alveolar macrophage (AM) polarization and subsequent fibroblast recruitment and activation not only in IPF but also in many other ILDs [22]. Finally, the 2022 study by Lin et al. evaluated pirfenidone’s effect on Tenon’s fibroblasts in vitro and observed a dose-dependent inhibition of fibroblast activity both in cytokine production and messenger ribonucleic acid (mRNA) levels [23]. Several trials have been conducted to assess pirfenidone’s efficacy in slowing the progression of IPF, validating its safety profile and ability to prolong the median survival rate of treated patients versus untreated [24]. A meta-analysis published in 2021 by Wu et al. examining nine different studies found the drug to increase the progression-free survival rate (PFS), reduce the frequency of exacerbations, and increase the overall survival rate [24]. Regardless of its promising effects on IPF progression, the main drawback of pirfenidone is its multiple side effects, the most common of which are mild to moderate gastrointestinal conditions and photosensitivity. Liver toxicity is another common side effect, with most patients reporting elevated levels of hepatic enzymes and rarely jaundice and liver failure [24,25,26]. In the ASCEND and CAPACITY studies, the two main registrational trials on pirfenidone, the discontinuation rate due to side effects was 15% overall (51 patients) in the group receiving pirfenidone treatment and 9% (30 patients) in the placebo group. Among these, five patients (>1%) stopped therapy due to the emergence of either skin rash or nausea. Upon combining the data from these studies, adverse events reported with a frequency exceeding 5% at the dosage of 2403 mg/day included nausea, dyspepsia, vomiting, anorexia, asthenia, skin rashes, photosensitization, and dizziness. Additionally, it was noted that gastrointestinal issues and photosensitization were dose-dependent and could be mitigated by administering the drug with food for the former and by minimizing sun exposure or using adequate sunscreen for the latter [27,28]. According to the technical data sheet, the recommended maintenance daily dose of Esbriet is three 267 mg capsules three times a day with food (2403 mg/day total). The maintenance dose should be reached over a 14-day titration period, for the first week: one capsule taken three times a day (801 mg/day); for the second week: two capsules taken three times a day (1602 mg/day); and from day 15 onwards: three capsules taken three times a day (2403 mg/day). In case of gastrointestinal, cutaneous, or hepatic adverse drug reactions (ADRs), the dose can be reduced (267 mg–534 mg) taken two to three times a day and then increased again up to the recommended daily dose. If symptoms persist, patients may be advised to discontinue treatment for 1–2 weeks to allow for resolution or, eventually, to discontinue treatment permanently [29]. In this study, we report our real-world experience regarding the efficacy and safety of pirfenidone in IPF patients, with a focus on the concomitant use of antithrombotic and anticoagulant drugs.

## 2. Results

In this two-center retrospective study, 55 patients with IPF were included, of whom 30 (54.5%) were males. The median (IQR) age at disease onset was 68.0 (10.0) years old, the median (IQR) age at baseline was 69.0 (10.8) years old, and 48 (87.3%) patients met the criteria for radiological diagnosis of IPF, while 7 (12.7%) underwent lung biopsy. At baseline, the median (IQR) FEV1, FVC, DLCO (% predicted), and 6MWD (meters) were 85.5 (30.5), 85.0 (28.5), 43.0 (25.5), and 451.5 (157.5), respectively. The baseline characteristics of our study population are reported in Table 1.

Overall, 16 (29.1%) patients underwent dose reduction, the median (IQR) daily adjusted dose was 801.0 (801.0) mg, and the median (IQR) time to dose reduction was 12.0 (42.5) months. Study treatment was discontinued prematurely in 20 (34.4%) patients, and the median (IQR) time to suspension was 18.0 (18.0) months. At a time span of 1 year after the introduction of pirfenidone, 7 (12.7%) patients continued treatment at a reduced dosage and 6 (10.9%) discontinued treatment. Overall, gastrointestinal intolerance and liver enzyme derangement were the two main causes of dose reduction (16.4% and 7.3%, respectively). Similarly, gastrointestinal intolerance and liver enzyme derangement were also the main causes of treatment suspension, followed by lung transplantation (9.1%, 9.1%, and 7.3%, respectively). Considering the 1-year observation period, all (6) dose reductions happened due to gastrointestinal intolerance, while treatment discontinuation happened due to gastrointestinal intolerance (3), lung transplantation (1), cutaneous disorders (1), and infection (1). Table 2 shows the causes of dose reduction and treatment discontinuation.

**Figure 1:**
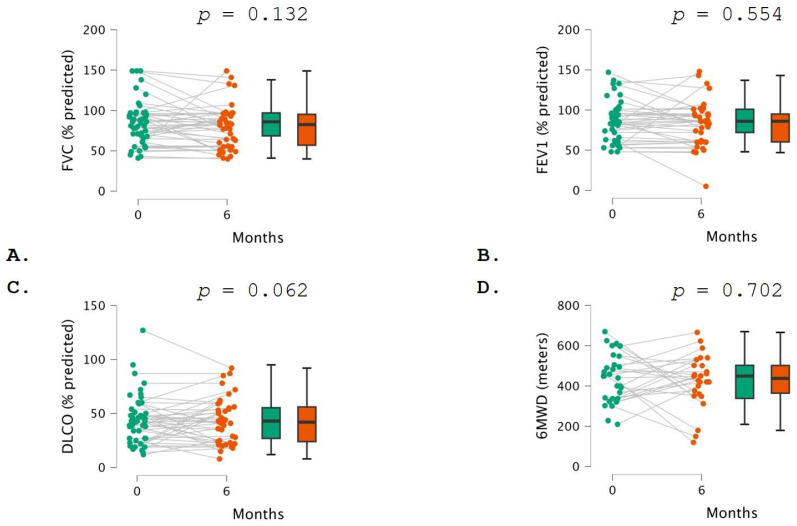
Comparison of lung function test measurements at baseline (green color) and 6 months (orange color). The two graphs represent (**A**) FVC, (**B**) FEV1, (**C**) DLCO, and (**D**) 6MWD. In the left part, each dot represents a patient, and each line reveals the same patient’s evolution over time. In the right part, data are presented as bar plots. Comparisons between pulmonary function test measurements and 6MWD at different time points were conducted using the paired *t*-test.

**Figure 2:**
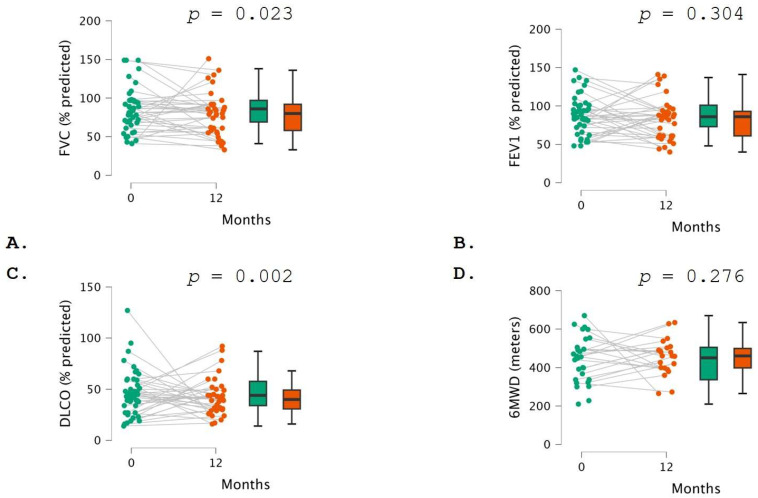
Comparison of lung function test measurements at baseline (green color) and 12 months (orange color). The two graphs represent (**A**) FVC, (**B**) FEV1, (**C**) DLCO, and (**D**) 6MWD. In the left part, each dot represents a patient, and each line reveals the same patient’s evolution over time. In the right part, data are presented as bar plots. Comparisons between pulmonary function test measurements and 6MWD at different time points were conducted using the paired *t*-test.

PFT did not show any significant change in FVC (*p* = 0.132), FEV1 (*p* = 0.554), DLCO (*p* = 0.062), or 6MWD (*p* = 0.702) between the baseline and 6 months following the initiation of pirfenidone (Table 3; Figure 1). Conversely, when comparing PFT measurements between the baseline and month 12, a statistically significant reduction was found in FVC (relative difference −6.0%; *p* = 0.023) and DLCO (relative difference −4.0%; *p* = 0.002). No significant changes in FEV1 (*p* = 0.733) and 6MWD (*p* = 0.542) were detected (Table 3; Figure 2).

Three (5%) patients reported one episode of bleeding in the groups receiving either concomitant anticoagulant or antithrombotic medications. Conversely, no bleeding was observed in the pirfenidone-alone group. In particular, we observed one episode of bleeding in the antiplatelet + pirfenidone group (1/10) and two bleedings in the anticoagulant + pirfenidone group (2/9). In conclusion, since we observed zero episodes of bleeding in the pirfenidone-alone group, the statistical significance cannot be evaluated. Interestingly, one patient on both anticoagulant and antiplatelet therapy did not present an episode of bleeding. The most reported adverse events were respiratory tract infections (36.4%), anorexia and/or decreased appetite (25.5%), diarrhea (18.2%), nausea (18.2%), and liver enzyme derangement (16.4%; Table 4). These events were generally mild to moderate and without clinical consequences, although, as previously reported in Table 2, some episodes led to dose adjustments or treatment discontinuation.

**Figure 3:**
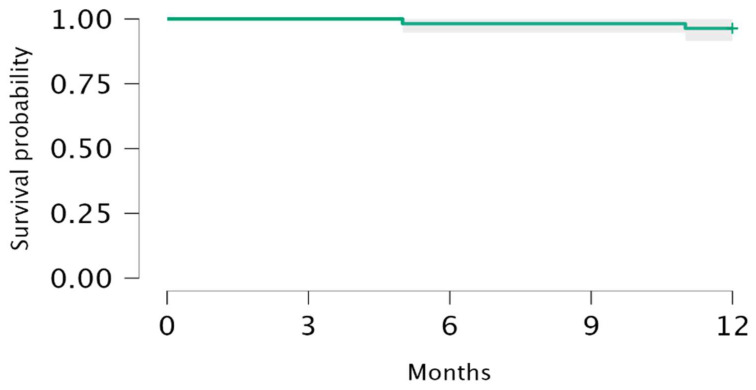
Kaplan–Meier estimates of the probability of survival at 12 months.

During the 1-year observation period, the overall survival was 98.2% (95% CI: 0.947–1.000) at 6 months and 96.4% (95% CI: 0.915–1.000) at 12 month since the introduction of pirfenidone (Figure 3).

## 3. Discussion

In this retrospective real-life observational study conducted in two centers that examined the efficacy and safety profile of pirfenidone in IPF, treatment with the study drug stabilized disease progression at 6 months, as measured by changes in FVC, FEV1, DLCO, and 6MWD, while it was associated with a statistically significant decline in FVC and DLCO at 12 months. Treatment with pirfenidone was generally safe, as most adverse events were of mild or moderate severity, although they led to discontinuation of treatment in the first year of therapy in 10.9% of patients. The most common adverse events were gastrointestinal and infectious. Data from our real-life study are in line with the main studies analyzed subsequently, both in terms of the percentage of patients who showed side effects and the types of side effects, and some of the side effects reported, such as dermatological or gastrointestinal problems, are manageable through prevention strategies and treatment as needed to ensure patient adherence to therapy [30]. In relation to the risk of bleeding, pirfenidone alone has not been associated with an equal or greater number of bleeding episodes compared to the concomitant use of anticoagulants or antiplatelet agents. Despite showing a significant reduction in the median percentage of predicted FVC and DLCO between the baseline and month 12, the relative differences were −6.0% (*p* = 0.023) and −4.0% (*p* = 0.002), respectively, both below the cutoffs generally accepted as indicators of disease progression [18,19]. Compared to the CAPACITY trials [28], although recorded at different time points, our results show a similar change in FVC (pooled population; week 72; mean change −8.5%) and DLCO (pooled population; week 72; mean change −8.8%) at 1 year. While these results generally confirm the efficacy of pirfenidone, the comparison is difficult to generalize due to the different nature of the studies, different sample sizes, and different follow-up times. Compared to some smaller, non-RCT, real-life works, the decline in FVC and DLCO showed a similar trajectory to ours, and the overall efficacy of pirfenidone in slowing IPF progression was reconfirmed [31,32]. Wuyts et al. reported a percentage predicted FVC 12-month relative difference of 0.9% (baseline vs. month 12, 81.2% vs. 82.1%) and a percentage predicted DLCO relative difference of −1.6% (baseline vs. month 12, 47.0% vs. 45.4%) [31]. Similarly, Majewski et al. reported a 12-month FVC relative difference of −0.68% and a DLCO relative difference of −4.94% [32]. With regard to adverse events, our data strengthen the safety profile of pirfenidone. In line with previous publications [28,31,32], gastrointestinal side effects (particularly nausea, diarrhea, and anorexia) were the most commonly reported, followed by respiratory tract infections and liver enzyme alterations. IRENE [33], an Italian multicenter, retrospective, observational study, examined real-world clinical data regarding IPF patients treated with pirfenidone. The investigator deemed 112 adverse events in 95 patients (25.1%) as related to pirfenidone. Skin disorders (14% of patients) and gastrointestinal reactions (12.4%) were the most frequently reported categories. Among skin-related adverse events, photosensitivity reactions (5.0% of patients), rash (3.7%), and erythema (3.2%) were most common, while nausea (3.7%), diarrhea (2.1%), and dyspepsia (2.1%) were prevalent gastrointestinal side effects. Nine serious adverse events in nine patients (2.4%) were considered pirfenidone-related by the investigator, including cases of erythema, pruritus, upper abdominal pain, gastroesophageal reflux disease, increased cholestasis enzymes (γ-glutamyltransferase) levels, increased hepatic enzyme levels, decreased appetite, and headache. Nine patients (2.4%) discontinued pirfenidone due to adverse events, but none were judged as related to pirfenidone. In 2020, Vietri et al. conducted an assessment of the effectiveness and safety profile of pirfenidone in a cohort of 91 patients with IPF treated at a single center in Italy between 2011 and 2019, involving a long-term follow-up period [34]. Their findings confirmed the favorable tolerability profile of the drug. Treatment-related adverse effects were documented in 25 patients (27%), primarily characterized as mild, with around half occurring within the initial 3 months of therapy. The most frequently reported adverse event was skin rash, affecting six patients (6.5%). Notably, only four patients permanently ceased treatment due to severe photosensitivity. Excluding these cases, temporary dose adjustments or interruptions due to cutaneous or gastrointestinal side effects were required in only 3 out of 87 patients (3.4%). No significant liver toxicity was observed. Gastrointestinal side effects were managed through pharmaceutical means, while broad-spectrum sunscreen was utilized to mitigate skin reactions. The study noted fewer adverse effects compared to similar real-world investigations. This improved tolerance may be attributed to thorough patient education regarding potential adverse drug reactions and the strong emphasis on implementing preventive measures. Differences in overall prevalence compared to the previously mentioned studies are probably due to different sample sizes. Emphasizing the mild or moderate severity of the reported adverse effects, in our study, fewer patients (10.9%) discontinued treatment compared to CAPACITY 004 (17%), CAPACITY 006 (18%), and Majewksi et al.’s work (adverse reactions and lung transplantation aggregated incidence, 16.9%) [28,32]. Overall, the use of pirfenidone was not associated with an increased risk of bleeding compared to the use of antithrombotic drugs. All recorded episodes occurred in the population undergoing concomitant antithrombotic treatment, and the overall incidence was comparable to a previous work by Glassberg et al. [35]. Similar results on the relationship between antifibrotic therapy and bleeding risk have already been reported for nintedanib [36,37,38,39]. The study offers a real-life perspective and provides a context closer to everyday clinical practice on the efficacy of pirfenidone, further validating its pivotal role in the treatment of IPF beyond registrational trials. Moreover, it better delineates the relationship between antifibrotic and antithrombotic therapy. Despite its strengths, it has some limitations. Firstly, the retrospective nature and small sample size (partly due to the low prevalence of IPF) make it difficult to generalize the results to a larger population of patients; in addition, the relatively high proportion of patients who discontinued treatment further reduced the study’s population, partly affecting the robustness of the statistical analysis. Secondly, missing data in repeated measurements and patients lost to follow-up could introduce a certain degree of bias in the assessment of lung function evolution, perhaps exacerbated by the limited number of study centers involved. Finally, the absence of a control group, as introduced in previous studies, limits the possibility of fully exploring the efficacy and safety profile of pirfenidone. We decided not to include a group of untreated patients, as a control, so as not to take away a therapeutic possibility for our patients. Despite all these limitations, we believe that managing patients with pulmonary fibrosis presents a daunting challenge for clinicians due to their complexity and frequent comorbidities, necessitating a multidisciplinary approach and meticulous care. Hence, our study aims to depict the intricate nature of this condition as encountered in clinical practice. In conclusion, our observational study in the real world of two reference centers for this rare pathology confirms the safety of the drug both in comorbid and very complicated patients, usually not included in clinical trials and registration studies. Furthermore, our work supports the safety of the use of pirfenidone even in patients treated with antithrombotic and anticoagulant agents.

## 4. Materials and Methods

### 4.1. Study Design and Population

This retrospective study included all adults diagnosed with IPF who started pirfenidone treatment between 2019 and 2022 at either study center (Department of Pulmonology, University Hospital of Cattinara, Trieste, Italy; Pulmonology Unit, General Hospital “Santa Maria degli Angeli”, Pordenone, Italy).

IPF diagnosis was made according to the 2018 ATS/ERS/JRS/ALAT Clinical Practice Guideline [10]. The assessment of ILD in our centers was based on HRCT and was conducted by radiologists possessing over a decade of expertise in lung imaging.

The initiation of therapy was considered the baseline. At the baseline, all the included patients started pirfenidone. We have accepted as the “starting dose” any daily dose achieved after the recommended 14-day titration; any negative variation from the starting dose has been considered as “dose reduction”. As per EMA/AIFA authorization, three 267 mg capsules three times a day (2403 mg/day total) was considered a full dose, and one to two capsules (267 mg–534 mg) two to three times a day were considered adjusted doses [27]. All patients underwent specialist discussion prior to the modification of the treatment protocol.

The inclusion criteria were: (a) radiologically or histologically confirmed diagnosis of IPF; (b) in treatment with pirfenidone; and (c) aged 18 years or older. The exclusion criteria were: (a) IPF diagnosis not meeting the 2018 ATS/ERS/JRS/ALAT Clinical Practice Guideline criteria [10]; (b) in concomitant treatment with immunosuppressants; (c) no available data regarding blood tests, diagnostic imaging, or histological results; (d) active cancer; or (e) diagnosed connective tissue diseases.

### 4.2. Data Collection

Clinical and functional data were retrospectively collected at the baseline and 6 and 12 months following the initiation of pirfenidone. The following clinical features were collected: sex, age at baseline, age at disease onset, body mass index (BMI), smoking history, pneumological comorbidities, pirfenidone therapeutic dose, tolerability and adverse events, changes in treatment protocol and time to change, mortality, and time to death, concomitant antithrombotic medications (anticoagulants and/or antiplatelets), bleeding episodes.

### 4.3. Pulmonary Function Tests

Lung function measurements were performed in accordance with ATS/ERS standards [40]. The collected parameters included forced expiratory volume in 1 s (FEV1), forced vital capacity (FVC), diffusing capacity of the lungs for carbon monoxide (DLCO), and 6 min walking test distance (6MWD). FVC, FEV1, and DLCO were described as a percentage of the predicted values for the patient’s age, sex, and height. A FVC decline > 10% and/or a DLCO decline > 15% over a period of 12 months was considered a clinically significant disease progression [18,19,30].

### 4.4. Statistical Analysis

The primary endpoint was a change in the percentage of predicted FVC, FEV1, DLCO, and 6MWD from the baseline to month 6 and from the baseline to month 12. Comparisons between pulmonary function test measurements at different time points were conducted using either the paired *t*-test or the non-parametric Wilcoxon signed-rank test when assumptions for the parametric test were not met. When comparing measurements at baseline to those at 6 months, the analysis only included individuals who had been on pirfenidone for a minimum of 4 months. Similarly, when comparing measurements at baseline and at 12 months, only individuals who had been on pirfenidone for at least 9 months were considered. The secondary endpoints were the incidence of bleeding events in the anticoagulants and antiplatelets populations and the prevalence of adverse events. Differences between study groups concerning categorical and dichotomous variables were evaluated using the chi-square test. Continuous variables are presented using either the mean (standard deviation) or the median (interquartile range) as appropriate, while categorical variables are expressed as the number (percentage). The normality of continuous variables was tested using the Shapiro–Wilk test. A survival analysis was conducted, taking the initiation of pirfenidone as the baseline and considering a 1-year observation period. The endpoint was defined as death from any cause, and the survival function was estimated using the Kaplan–Meier method. A two-tailed *p*-value <0.05 was considered statistically significant. All analyses were conducted using JASP software (version 18.3).

## 5. Conclusions

In this real-life study conducted in two Italian referral centers, pirfenidone for the treatment of IPF was associated with non-significant functional decline at both 6 and 12 months after the start of therapy, effectively stabilizing disease progression. Furthermore, these observations confirm the safety profile of pirfenidone, even in a population treated with antithrombotic and/or anticoagulant drugs. However, further research is underway to validate these findings.

## Figures and Tables

**Table 1:** Baseline characteristics of the study population.

Characteristics	*n* = 55
Age (years)	69.0 (10.8)
Disease onset (years)	68.0 (10.0)
Gender	
Males	30 (54.5)
Females	25 (45.5)
BMI, mean (SD)	28.4 (4.6)
Ever-smoker	36 (65.5)
Diagnosis	
HRCT	48 (87.3)
Histological	7 (12.7)
Previous coexisting lung disease	
COPD	2 (3.6)
Emphysema	4 (7.3)
OSA	3 (5.5)
Pulmonary hypertension	3 (5.5)
Concomitant antithrombotic medications	
Anticoagulants	10 (18.2)
Antiplatelets	11 (20.0)
Pulmonary function tests	
FEV1 (% predicted)	85.5 (30.5)
FVC (% predicted)	85.0 (28.5)
DLCO (% predicted)	43.0 (25.5)
6MWD (m)	451.5 (157.5)

Data are presented as No. (%) or median (IQR), unless otherwise stated. BMI: body mass index; HRCT: high-resolution computed tomography; COPD: chronic obstructive pulmonary disease; OSA: obstructive sleep apnea; FEV1: forced expiratory volume in 1 s; FVC: forced vital capacity; DLCO: diffusing capacity of the lungs for carbon monoxide; 6MWD: 6 min walking distance.

**Table 2:** Completion of therapy and causes for treatment protocol modification.

Dose Reduction *	16 (29.1)
Gastrointestinal intolerance	9 (16.4)
Liver enzymes derangement	4 (7.3)
**Treatment suspension ‡**	**20 (34.4)**
Transplantation	4 (7.3)
Gastrointestinal intolerance	5 (9.1)
Cutaneous disorders	1 (1.8)
Dizziness	1 (1.8)
Inefficacy	1 (1.8)
Liver enzymes derangement	5 (9.1)
Arrhythmia	1 (1.8)
Infection	1 (1.8)

Data are presented as No. (%). * Three patients did not report any cause. ‡ One patient did not report any cause.

**Table 3:** Comparison of lung function test measurements after 6 and 12 months.

Baseline	*n*	Six Months	*n*	*p*	Baseline	*n*	Twelve Months	*n*	*p*
FVC (% predicted)									
86.0 (28.5)	47	82.5 (38.3)	42	0.132	86.0 (28.0)	45	80.0 (34.0)	37	0.023
FEV1 (% predicted)									
86.0 (29.0)	45	86.0 (34.8)	38	0.554	86.0 (28.0)	43	86.0 (32.0)	35	0.304
DLCO (% predicted)									
43.0 (28.3)	48	42.0 (32.0)	41	0.062	44.0 (23.8)	46	40.0 (18.5)	36	0.002
6MWD (meters)									
449.0 (164.5)	30	437.5 (137.8)	26	0.702	450.0 (168.0)	29	460.0 (100.5)	22	0.276

Data are presented as median (IQR). When comparing measurements at the baseline and 6 months, the analysis only included individuals who had been on pirfenidone for a minimum of 4 months; when comparing measurements at baseline and 12 months, the analysis only included individuals who had been on pirfenidone for a minimum of 9 months. FVC: forced vital capacity; FEV1: forced expiratory volume in 1 s; DLCO: diffusing capacity of the lungs for carbon monoxide; 6MWD: 6 min walking distance.

**Table 4:** Treatment-associated adverse events.

	*n* = 55
Diarrhea	10 (18.2)
Nausea	10 (18.2)
Anorexia/decreased appetite	14 (25.5)
Liver enzymes derangement	9 (16.4)
Jaundice	0 (0.0)
Photosensitivity	7 (12.7)
Rash	2 (3.6)
Respiratory tract infection	20 (36.4)
Urinary tract infection	2 (3.6)
Anaphylaxis	0 (0.0)
Headache	4 (7.3)
Dizziness	4 (7.3)
Hot flush	2 (3.6)
Insomnia	0 (0.0)
Arthralgia	0 (0.0)

Data are presented as No. (%).

## Data Availability

Deidentified participant data will be made available upon motivated request to the Corresponding Author.
